# Thermodynamic Properties, Crystallization Kinetics and Crystal Morphology of Plutonium Oxalate Crystals: A Review

**DOI:** 10.3390/molecules31091391

**Published:** 2026-04-23

**Authors:** Yunhai Huang, Yongxue Guo, Siwen Yuan, Guanchen Zhou, Lei Li, Xuefeng Hou, Dehui Wu, Hongxun Hao, Yantao Hu

**Affiliations:** 1 China Nuclear Power Engineering Co., Ltd., Beijing 100840, China; yunhaihuang@tju.edu.cn (Y.H.); guoyx@cnpe.cc (Y.G.); yuansw@cnpe.cc (S.Y.); zhougc@cnpe.cc (G.Z.); lileia@cnpe.cc (L.L.); houxf@cnpe.cc (X.H.); wudh@cnpe.cc (D.W.); 2 National Engineering Research Center of Industrial Crystallization Technology, School of Chemical Engineering and Technology, Tianjin University, Tianjin 300072, China; hongxunhao@tju.edu.cn

**Keywords:** plutonium oxalate, solubility, crystallization kinetics, crystal morphology

## Abstract

As a key type of precursor material in the nuclear fuel cycle process, plutonium oxalate has long played a critical role in the purification and conversion of plutonium. Its crystallization behavior directly affects the subsequent production process and properties of plutonium oxide. This review systematically summarizes the research progress of plutonium oxalate crystals in thermodynamics, crystallization kinetics, and crystal morphology. It introduces the structural characteristics of plutonium oxalate crystals, their solubility in nitric acid-oxalic acid mixed systems, and the thermodynamic properties such as the redox stability of plutonium oxalate crystals of different valence states. It also summarizes the nucleation, growth, and coprecipitation kinetics of plutonium oxalate crystals. The diversity of plutonium oxalate crystal morphologies and their influence on subsequent thermal decomposition are discussed.

## 1. Introduction

Plutonium (Pu) oxalate (especially Pu(IV) oxalate) is an indispensable key material in the nuclear fuel cycle process. It has been used for a long time in the purification and conversion of plutonium, and the treatment of radioactive waste. Since the 1940s, the process of preparing plutonium oxide by thermal decomposition of plutonium oxalate, with the “plutonium(IV) oxalate method” as the core, has been widely used in the nuclear industry and has become the standard production process for global nuclear fuel reprocessing plants [1,2,3]. This process is simple to operate and stable, and has been widely applied for a long time. However, the underlying physical and chemical mechanisms, especially the structural evolution and phase transition process of plutonium oxalate, have not yet been fully elucidated [4,5]. This deficiency in basic knowledge has restricted the further optimization of the plutonium conversion process.

In recent years, thanks to the advancements in characterization techniques such as high-resolution Raman spectroscopy, infrared spectroscopy, in situ X-ray diffraction, and scanning electron microscopy, as well as the development of computational chemistry methods like density functional theory (DFT), significant progress has been made in the study of the plutonium oxalate system [6,7,8,9,10,11]. Researchers have been able to systematically investigate its crystallization process, thermal decomposition behavior, and the evolution of microscopic morphology at the molecular and atomic scales [12,13,14]. These efforts not only filled the long-standing gap in vibrational spectroscopy and crystal structure data for this system but also provided new perspectives for a deeper understanding of coordination behavior and phase transformation mechanisms in complex chemical environments [15,16]. It is worth noting that these studies are particularly challenging due to the radiological hazards associated with plutonium, which necessitate specialized handling protocols, remote-controlled experimental setups, and rigorous radiation protection measures. Consequently, the acquisition of high-resolution spectroscopic and diffraction data, as well as the in situ characterization of crystallization and decomposition processes under such constrained conditions, represents a substantial technical achievement. This article aims to review and summarize the latest research progress in the thermodynamics, crystallization kinetics, and crystal morphology of plutonium oxalate, providing a more solid scientific basis and technical support for the efficient operation of the nuclear fuel cycle and the safe disposal of radioactive waste.

## 2. The Thermodynamics of Plutonium Oxalate Crystals

### 2.1. Crystal Structure

Understanding the basic structure of crystal materials is crucial for their subsequent application [17,18,19]. For some crystal materials with a poor single crystal quality, which are difficult to obtain as single-crystal structures through experiments, density functional theory calculations have become a powerful tool to reveal their electronic structure and bonding nature, providing microscopic information that is difficult to obtain through experimental means at the atomic level [20,21,22]. Some studies have inferred the crystal structure of Pu(C_2_O_4_)_2_·6H_2_O through computational simulation. Pu(C_2_O_4_)_2_·6H_2_O has a layered monoclinic crystal structure, which is isomorphic to the oxalates of tetravalent uranium and neptunium [9,23,24]. Inside the crystal, plutonium ions have two different local coordination environments with coordination numbers of 8 and 9, reflecting a slight distortion of the structure. The analysis of its electronic structure indicates that the material is in an antiferromagnetic ground state, and there is a weak hybridization between the 5f electron orbitals of plutonium and the 2p orbitals of the ligand oxygen, presenting an overall bonding characteristic dominated by ionic bonds. The Bader charge calculation based on the topological analysis of the electron density shows that the effective positive charge carried by the plutonium atom is approximately +2.5*e*, further confirming the ionic nature of its bonding [9]. These theoretical research results provide a theoretical basis for understanding the crystallization process of plutonium(IV) oxalate and predicting the structure and stability of possible intermediates generated during the thermal decomposition of plutonium(IV) oxalate.

**Figure 1:**
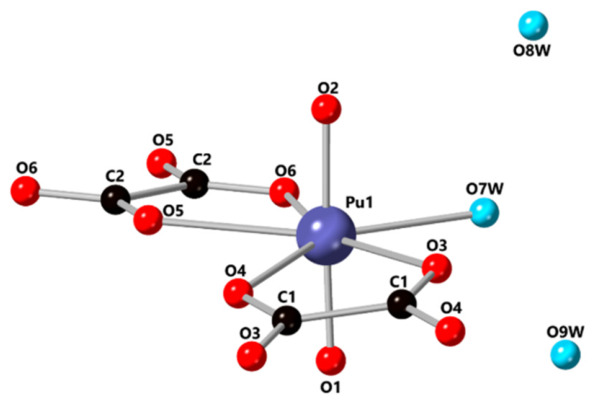
Ball-and-stick representation of [PuO_2_(C_2_O_4_)(H_2_O)]·2H_2_O with atoms labeled. H atoms were removed for clarity [25].

Some researchers have determined the crystal structure of [PuO_2_(C_2_O_4_)(H_2_O)]·2H_2_O through experiments [25]. The unit cell of it is *a* = 5.5993(3) Å, *b* = 16.8797(12) Å, *c* = 9.3886(6) Å, and *β* = 98.713(6)° in space group *P*2_1_/*c*. Plutonium(VI) is present as the plutonyl ion (PuO_2_^2+^, O=P=O) with an O=Pu=O bond angle of 177.8(5)°. The plutonyl Pu=O bond lengths are 1.732(9) and 1.745(9) Å, consistent with the expected bond length for the +6 oxidation state (1.75 Å). The labeled structure of [PuO_2_(C_2_O_4_)(H_2_O)]·2H_2_O is shown in Figure 1.

### 2.2. Solubility

**Figure 2:**
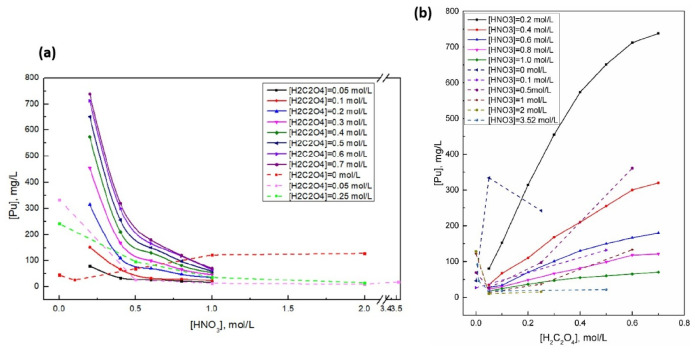
The solubility of plutonium oxalate (IV) in nitric acid-oxalic acid mixed solvents at different concentration ranges [26,27]. (**a**) The solubility changes with the concentration of nitric acid; (**b**) The solubility changes with the concentration of oxalic acid.

The crystallization process of plutonium oxalate is closely related to its dissolution behavior in solution. For the solution system of the reaction between plutonium nitrate and oxalic acid, the solubility of plutonium(IV) oxalate crystals is jointly affected by the acidity of the system and the concentration of oxalate ions. In the mixed solution of nitric acid and oxalic acid, the solubility of plutonium(IV) oxalate does not monotonically change with the concentration of oxalic acid or nitric acid. According to the current research on the solubility of plutonium(IV) oxalate in some specific concentration ranges of nitric acid and oxalic acid, it is indicated that in the mixed solution of nitric acid and oxalic acid, within the range of 0.5–3.52 mol/L for nitric acid concentration and 0–0.6 mol/L for oxalic acid concentration, the solubility of plutonium(IV) oxalate first decreases and then increases with the increase in oxalic acid concentration; within the range of 0–6 mol/L for nitric acid concentration and 0–0.1 mol/L for oxalic acid concentration, the solubility of plutonium(IV) oxalate first decreases and then increases with the increase in nitric acid concentration [26,27]. The solubility of plutonium(IV) oxalate in nitric acid-oxalic acid mixed solvents at different concentration ranges is shown in Figure 2.

The plutonium(IV) oxalate exhibits a low solubility in oxalic acid, but its solubility does not monotonically change with the concentration of oxalic acid. At a low oxalic acid concentration, plutonium(IV) mainly precipitates as the solid form of Pu(C_2_O_4_)_2_·6H_2_O, and its solubility decreases as the oxalic acid concentration increases [28]. However, when the oxalic acid concentration exceeds a certain threshold, plutonium(IV) tends to coordinate with excess oxalate ions, forming soluble complex anions, which leads to an increase in its solubility and presents a characteristic “low point” on the solubility–concentration curve [27,28]. This phenomenon is crucial for the design of nuclear waste treatment processes, such as determining the composition of the crystallization endpoint solvent for plutonium(IV) oxalate crystals.

### 2.3. Redox Stability

**Figure 3:**
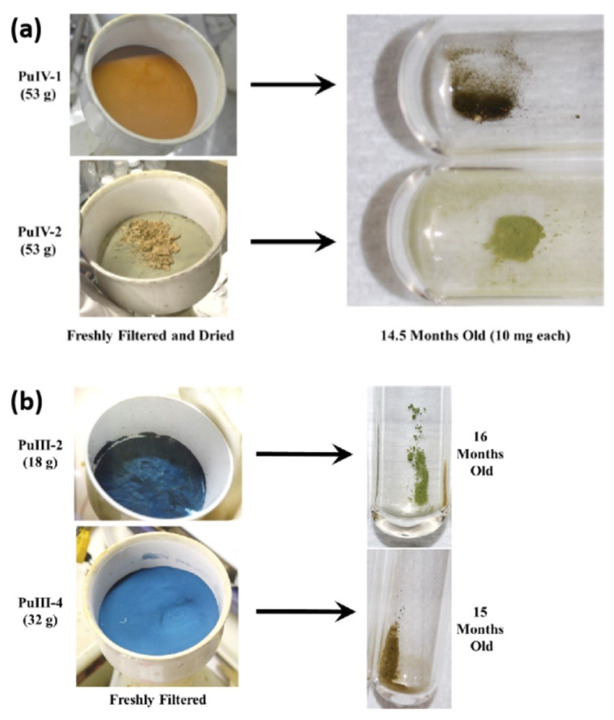
Photographs of different batches of plutonium(III) and plutonium(IV) oxalate freshly filtered (**left**) and aged in air at room temperature (**right**) [32]. (**a**) Plutonium(IV) system; (**b**) Plutonium(III) system.

The plutonium element exists in multiple oxidation states in the oxalic acid system. The redox stability of different valence states of oxalate salts directly affects the purity of the crystalline product and the subsequent processing behavior [29,30,31]. Studies have found that crystallization under specific acidic conditions can yield plutonium(VI) oxalate [PuO_2_(C_2_O_4_)(H_2_O)]·2H_2_O, but it is extremely sensitive to light and will rapidly undergo photoreduction reactions under environmental light exposure, generating plutonium(IV) oxalate and other valence state oxalate mixtures [25]. This indicates that plutonium(VI) is unstable in the oxalic acid medium, so it is necessary to avoid light during the crystallization operation of plutonium(VI) oxalate to prevent the occurrence of reduction reactions. Plutonium(IV) oxalate is relatively stable under acidic conditions, but it still undergoes complex oxidation–reduction reactions during the thermal decomposition process, such as releasing carbon dioxide and generating intermediate products such as plutonium oxycarbonate [15]. Some researchers have studied the solid-state self-degradation process of plutonium(III) and plutonium(IV) oxalate at room temperature. The results show that even if the preparation and storage methods of the materials are the same, different batches of plutonium oxalate may form decomposition products of different colors during aging. The analysis results of powder X-ray diffraction (PXRD), infrared spectroscopy and near-infrared spectroscopy indicate that plutonium(III) and plutonium(IV) oxalate may undergo self-degradation reactions in the air at room temperature, forming similar decomposition products, which are very likely to be PuO_2_ nanocrystals [32]. Photographs of different batches of plutonium(III) and plutonium(IV) oxalate freshly filtered (left) and aged in air at room temperature (right) are shown in Figure 3.

## 3. Crystallization Kinetics of Plutonium Oxalate

### 3.1. Nucleation and Growth Kinetics

**Figure 4:**
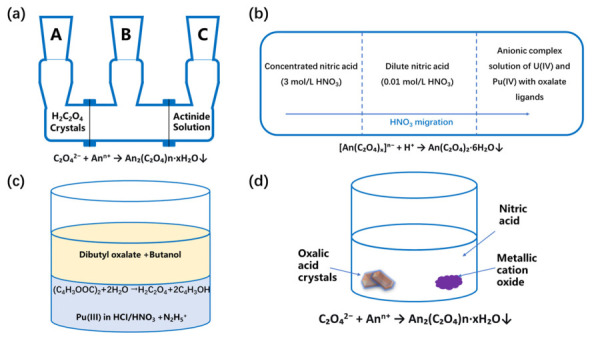
The experimental illustrations and corresponding mechanisms of the methods to gain plutonium oxalate single crystals [45,46]. (**a**) method 1; (**b**) method 2; (**c**) method 3; (**d**) method 4.

The particle size distribution of crystals has a significant impact on subsequent operations such as filtration [33,34,35,36]. The nucleation and growth kinetics of the crystallization process play a crucial role in the particle size distribution of crystal products [37,38], and the supersaturation of the solution is a key factor affecting the crystallization kinetics [39,40,41]. Since the traditional rapid precipitation method involves instant mixing of the plutonium nitrate solution and oxalic acid, it leads to a high local supersaturation, which easily causes burst nucleation and generates a large number of fine crystals, which is not conducive to obtaining larger-sized crystal products [42,43,44], and it is even more difficult to obtain single crystals that can be used for analyzing crystal structures. Therefore, some researchers have obtained plutonium oxalate single crystals through some special experimental methods. The first method is based on the slow diffusion technology of a three-compartment diffusion cell. By controlling the contact between oxalic acid and the Pu-Am solution through a compressed glass fiber membrane, single crystals of Pu-Am oxalate were prepared [45]. The second method utilized soluble anion complexes that decomposed during the slow acidification process to prepare U-Pu oxalate single crystals [45]. The third method employed the slow hydrolysis of oxalate esters in an acidic medium to generate oxalic acid, thereby controlling the crystallization rate of plutonium oxalate, and obtained single crystals [46]. The fourth method used solid plutonium oxide (such as PuO_2_) as the precursor, which is slowly transformed in the mixed solution of dilute nitric acid and oxalic acid to directly produce plutonium oxalate crystals [46]. These methods effectively avoided rapid precipitation by controlling the release rate of reactants and local supersaturation, and successfully obtained plutonium oxalate single crystals suitable for structural analysis. The experimental illustrations and corresponding mechanisms of the four methods are shown in Figure 4.

**Figure 5:**
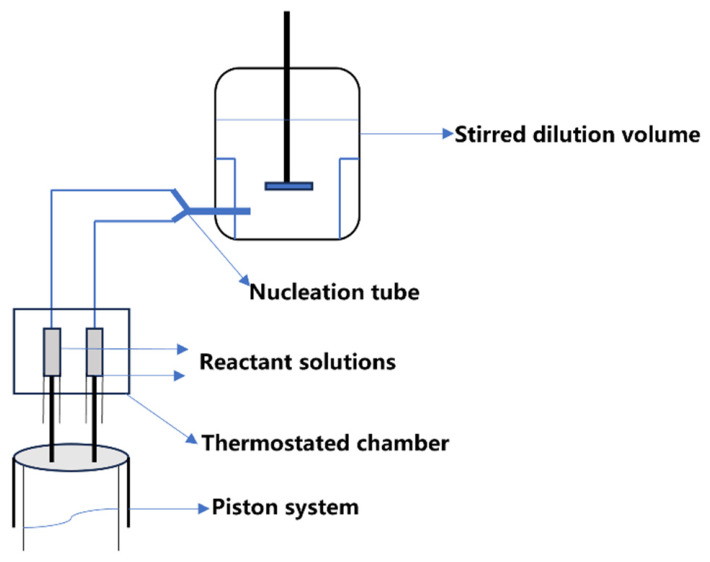
The basic operating principle of the experimental setups to measure the nucleation and growth kinetics of plutonium(IV) oxalate.

Some researchers designed specialized experimental setups to measure the nucleation and growth kinetics of plutonium(IV) oxalate by controlling the dilution ratio [47]. The basic operating principle of the apparatus is schematically shown in Figure 5. This method effectively suppressed the dissolution of crystal nuclei, secondary nucleation, and crystal agglomeration by keeping the initial apparent supersaturation *S*_N_ within the range of 1.66–1.67, and thus obtained reliable kinetic parameters. Their results indicated that within the temperature range of 25–50 °C and the supersaturation range of 8.37–22.47, the nucleation kinetic equation of plutonium(IV) oxalate is $R_{N}=4.8\times 10^{23}exp(-36.2/RT)exp[-20.2/(lnS{)}^{2}]$, where *R_N_* represents the number of nuclei formed per unit volume and time, *S* represents the supersaturation ratio, *R* represents the ideal gas constant, *T* represents the absolute temperature. The growth kinetics equation is $G=7.3\times 10^{-7}exp(-25.7/RT)(c-c_{\mathrm{eq}}{)}^{1.1}$, where *G* represents the linear crystal growth rate, *c* and *c*_eq,_ respectively, represent initial and equalizing concentration.

### 3.2. Co-Precipitation Kinetics

When dealing with nuclear waste liquid, it is sometimes necessary to perform co-precipitation of plutonium with other coexisting actinide elements (such as americium), and the separation efficiency and the purity of the final product are directly influenced by the co-precipitation kinetics behavior [28,48,49]. Studies have shown that in a mixed solution of nitric acid and oxalic acid, Pu(IV) and Am(III) can form a co-precipitate with bismuth oxalate. The rate and completeness of this process are simultaneously affected by three factors: the acidity of the solution, the concentration of oxalic acid, and the concentration of bismuth ions. When the acidity is low (such as 0.5 mol/L nitric acid), the co-precipitation reaction proceeds more rapidly and completely, and equilibrium is usually reached within 3 h. Conversely, when the acidity is high (such as 3.75 mol/L nitric acid), the precipitation rate slows down significantly, and the precipitation is incomplete. By establishing a co-precipitation kinetics model for analysis, the supersaturation of the precipitant (bismuth) is the key factor determining the co-precipitation efficiency [28]. The research on co-precipitation kinetics provides a design basis for efficient and selective separation of plutonium and other actinide elements from complex nuclear waste liquid.

## 4. The Crystal Morphology of Plutonium Oxalate

### 4.1. The Diversity of Crystal Morphology

**Figure 6:**
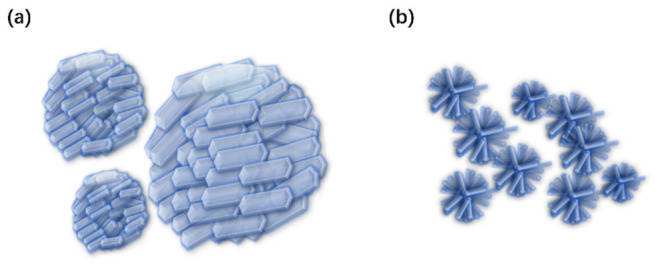
Schematic diagrams of the morphology of plutonium oxalate crystals with different precipitation methods. (**a**) direct strike synthesis; (**b**) reverse strike synthesis.

The crystal morphology of plutonium oxalate is jointly influenced by multiple factors, including precipitation mode, valence state, and the chemical environment of the solution. The precipitation method can have a significant impact on the morphology and properties of crystals [50,51,52]. Studies have shown that the oxalate from direct strike synthesis (adding oxalic acid to plutonium solution) is prone to obtaining spherical or nearly spherical plutonium oxalate agglomerated particles, which are usually composed of smaller, more regularly shaped primary particles. The oxalate from reverse strike synthesis (adding plutonium solution to oxalic acid) is more likely to obtain irregular, loose and porous aggregates, which often have large microcrack networks running through the entire particle, indicating a higher degree of agglomeration and less stable structure [14]. Schematic diagrams of the morphology of plutonium oxalate crystals with different precipitation methods are shown in Figure 6.

In addition, studies have shown that when a solution of plutonium(IV) nitrate is slowly dripped into a stirred oxalic acid solution at 25 °C, the resulting Pu(IV) oxalate hexahydrate particles exhibit a complex mixed morphology, including cubic crystalline, irregular crystals, and amorphous substances. The particle surfaces are sub-euhedral, with sub-angular edges, low sphericity, and generally present surface cracks and edge erosion [15]. Further, high-quality single crystals with consistent morphology can be obtained through an appropriate crystal growth method, which can be used to reveal the correlation between the intrinsic crystal structure and morphology [45]. Studies have shown that the morphology of plutonium oxalate is influenced by its oxidation state and charge-balancing ions in the system. In a mixed system of Pu(III)/Pu(IV), the concentration of single-charge cations such as hydrazine can directionally control the crystal phase of the crystal products. In a system with high hydrazine concentration, the hexagonal crystal phase of Pu(IV) oxalate is favored to form; in a system with medium hydrazine concentration, the tetragonal crystal phase of Pu(IV) oxalate is more likely to form; and in a system with low hydrazine concentration, the monoclinic crystal phase dominated by Pu(III) oxalate is easily formed. Single crystals of different crystal phases exhibit different macroscopic morphologies and colors, indicating that the internal crystal structure of plutonium oxalate affects its growth mechanism and, consequently, its macroscopic morphology [45].

In summary, the morphology of plutonium oxalate is a multi-scale characteristic system: from the macroscopic particle shape and agglomeration state determined by the precipitation process to the intrinsic crystal structure and phase influenced by the crystal growth process. These multi-scale morphological features affect its subsequent filtration process and the physicochemical behavior during thermal treatment to convert it into plutonium oxide. More importantly, they serve as “process fingerprints”, recording the entire chain of information from solution chemistry, precipitation conditions, to thermal treatment. Therefore, the systematic characterization and understanding of the morphology of plutonium oxalate is of crucial significance for the optimization of nuclear fuel cycle processes.

### 4.2. The Relationship Between the Crystal Morphology of Plutonium Oxalate and Its Thermal Decomposition

**Figure 7:**
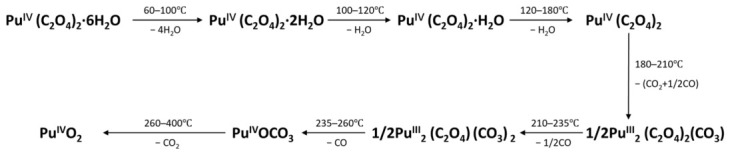
Mechanism of Pu(IV) oxalate decomposition [4].

The thermal decomposition process of Pu(IV) oxalate in the air is shown in Figure 7 [4].

The thermal decomposition process and the characteristics of the final product of plutonium oxalate are closely related to its crystal morphology [6,53,54]. A profound understanding of this relationship is crucial for regulating the performance of the final plutonium oxide product. As mentioned in Section 3.1, different precipitation methods yield plutonium oxalate crystals with distinct macroscopic morphologies (such as spherical or irregular aggregates), and these morphologies remain unchanged during the subsequent thermal decomposition process of plutonium oxalate, demonstrating a “memory effect”. During the thermal decomposition of plutonium oxalate in the air and its conversion into plutonium oxide, although there were intense physical and chemical changes within the particles (such as dehydration, decomposition of the oxalate ligands, and gas release), which significantly increased the volume proportion of internal pores and microcracks, the external macroscopic morphology of the particles remained largely unchanged. The pores and micro-crack networks formed within the plutonium oxalate crystal lumps provided channels for the release of the gases produced during decomposition. Therefore, the particles maintained their skeletal structure and retained the morphology of the plutonium oxalate during the intense mass loss process.

Analysis of the SEM images and corresponding Raman spectra during the thermal decomposition of plutonium oxalate reveals that oxalate crystals with rough surfaces and developed pore structures exhibit higher reactivity during the decomposition process [15]. This may be due to their larger specific surface area, which facilitates the diffusion of reaction gases and makes it easier to generate nano-sized plutonium oxide particles. Conversely, if the structure of the aggregated plutonium oxalate crystals is dense and the size is large, the thermal decomposition rate may be relatively slow, and the restricted heat and mass transfer can easily lead to the residual carbon impurities inside the particles [15]. Additionally, the irregularity of the crystal morphology may cause uneven heat distribution during the thermal decomposition process, which may result in the crystals in different regions decomposing at different times and potentially affect the consistency of the product. Even after high-temperature heat treatment at 450 °C, residual carbon content up to 5000 ppm can still be detected in the final product [55]. These carbon residues mainly originate from the incomplete decomposition of oxalate ligands, and their residual amount is closely related to the morphology and structure of the initial oxalate particles [15]. Therefore, actively controlling the morphology of oxalate during the crystallization step is an effective way to reduce carbon impurities in the final oxide and improve the purity of the plutonium oxide product.

## 5. Conclusions

The crystallization of plutonium oxalate is a complex system that integrates thermodynamics, kinetics, and morphological evolution. Although current research has made certain progress in aspects such as crystal structure analysis, thermodynamic and kinetic mechanisms, and coprecipitation separation behavior, it still faces some challenges. At the thermodynamic level, although there is a basic understanding of the dissolution behavior of Pu(IV) oxalates, the relative stability of different valence state plutonium oxalates in complex solution environments and the competitive coordination mechanisms still require more in-depth exploration. In the field of morphology control, how to precisely regulate the morphology of oxalate crystals by introducing additives and optimizing crystallization conditions, and to quantitatively establish the structure–activity relationship between precursor morphology and final oxide performance, are key scientific issues that need to be addressed urgently.

## 6. Future Directions

**Figure 8:**
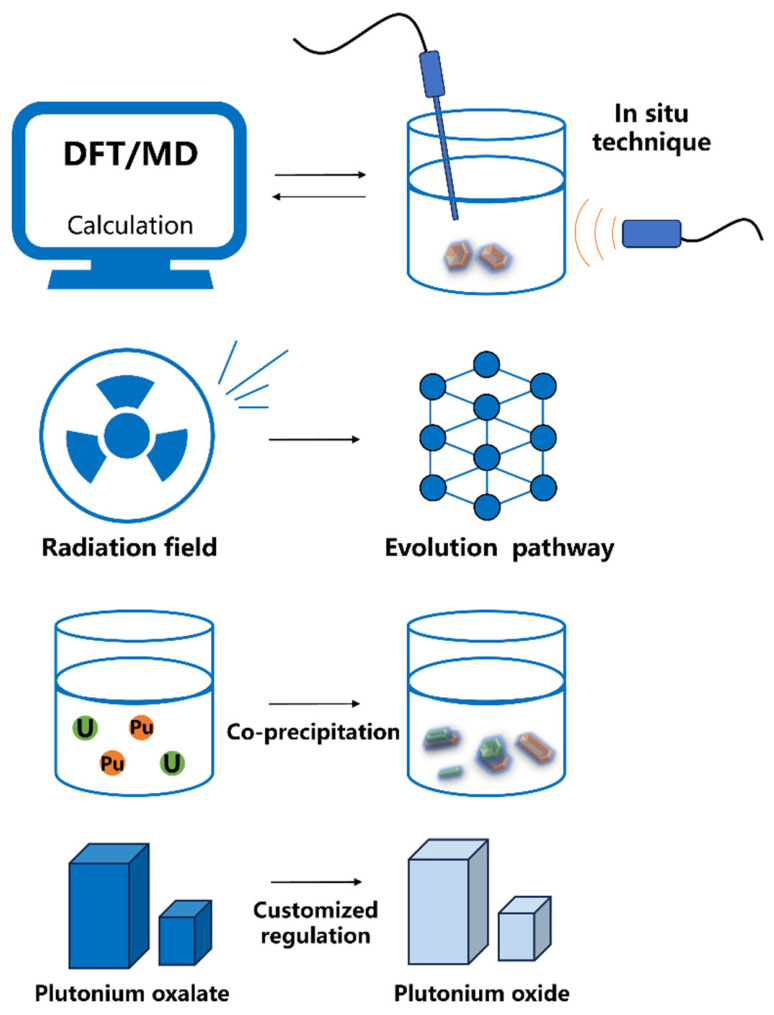
Schematic diagram of the research idea for the crystallization process of plutonium oxalate.

In the future, the research on the crystallization process of plutonium oxalate can be further deepened in the following directions (Figure 8).

Firstly, theoretical calculations and experimental research should be further strengthened to complement each other. Methods such as DFT calculations and molecular dynamics should be used to simulate the coordination structure, nucleation process and growth process of plutonium oxalate in solution, and in situ and different working conditions experimental techniques should be developed to verify the theoretical predictions. Secondly, the impact of radiation fields and photochemical effects in actual nuclear environments on the crystal of plutonium oxalate should be highly emphasized, and its evolution behavior under radiation conditions should be evaluated. Thirdly, at the engineering application level, the separation process of co-precipitation and other separation methods should be continuously optimized, and more selective and efficient separation methods should be developed to achieve safe and thorough separation of plutonium and other actinide elements from nuclear waste. Finally, through the development of cross-scale morphology control technology, a specifically designed precursor of plutonium oxalate should be prepared to achieve “customized” regulation of the physical and chemical properties of the final plutonium oxide product, so as to meet diverse application requirements. Continuous in-depth research on the crystallization and transformation process of plutonium oxalate will not only deepen the understanding of the solid chemical laws of actinide elements but also lay a more solid scientific and technical foundation for the efficient recycling of nuclear fuel, the safe disposal of radioactive waste, and the progress of nuclear security supervision technologies.

## Data Availability

No new data were created or analyzed in this study.
